# Supplementary material for: Metabolomics, Genetics, and Environmental Factors: Intersecting Paths in Abdominal Aortic Aneurysm
Source: Int J Mol Sci. 2025 Feb 11;26(4):1498. doi: 10.3390/ijms26041498 (PMC11855682; doi:10.3390/ijms26041498)
Supplement: Supplementary file 1 [file ijms-26-01498-s001.zip › Supplemental Figure S1.pdf]

Supplemental Figure S1: Genetic regulation of metabolites and their impact on abdominal aortic aneurysm progression.

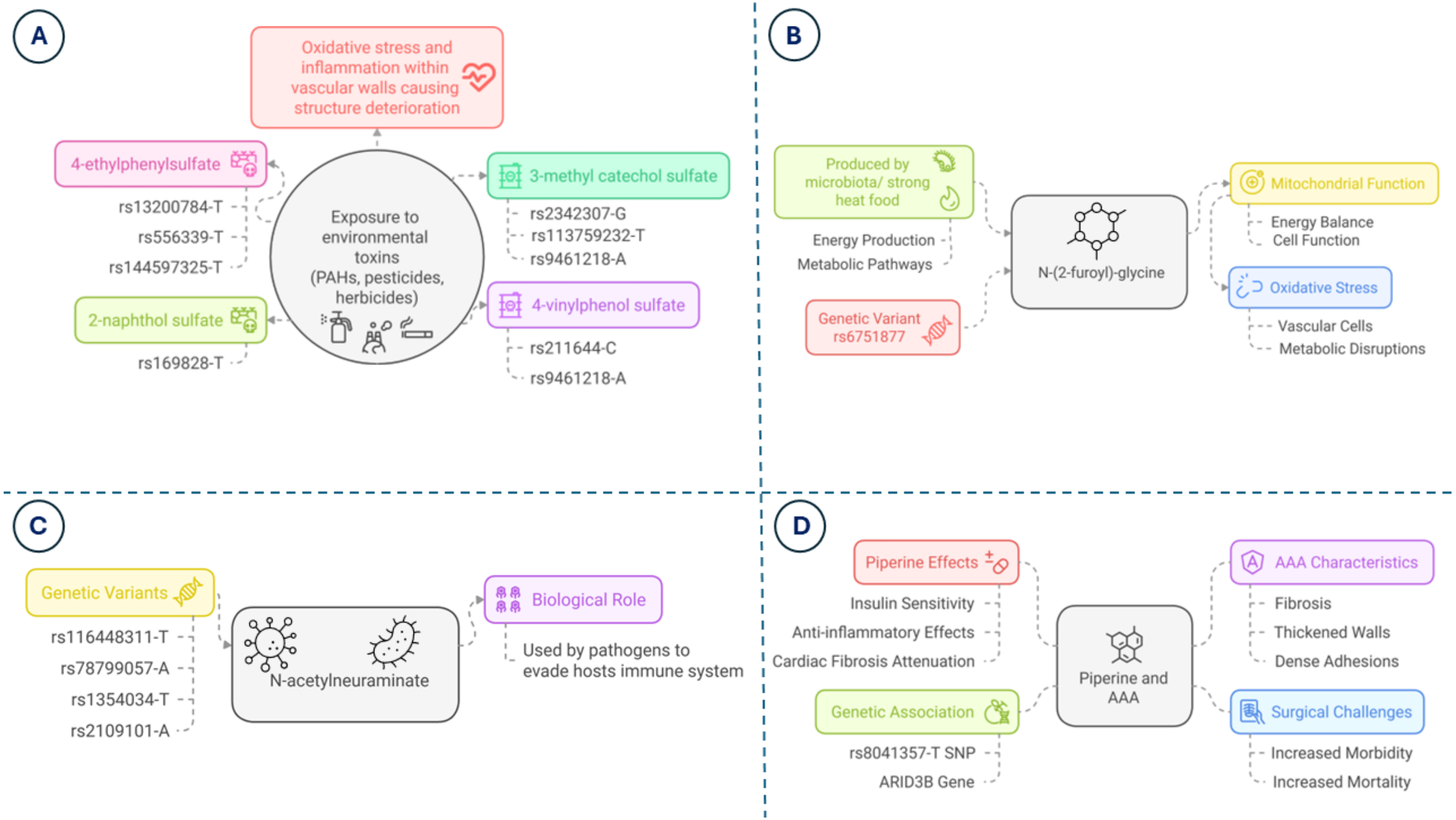

A. Xenobiotics linked to AAA development through increased oxidative stress and inflammation, influenced by environmental toxins like PAHs, pesticides, and herbicides. **B.** N-(2-furoyl)glycine, associated with fatty acid catabolism, may disrupt mitochondrial function and contribute to the energy imbalances in vascular cells, exacerbating AAA. **C.** N-acetylneuraminate, a sialic acid crucial for cell communication and immune response, and is used by pathogens to evade the immune system. **D.** Piperine, with its potent antiinflammatory effects and cardiac fibrosis attenuation, typically counters thickened walls and dense adhesions in vascular tissues; its decreased levels in AAA patients suggest a reduction in vascular protective mechanisms.
